# Caring for a child with a lower respiratory tract infection: Parents’ perspectives

**DOI:** 10.4102/sajp.v82i1.2251

**Published:** 2026-02-14

**Authors:** Danielle Foot, Joanne L. Potterton

**Affiliations:** 1 Department of Physiotherapy, Faculty of Health Sciences, University of the Witwatersrand, Johannesburg, South Africa

**Keywords:** chest physiotherapy, children, lower respiratory tract infection, medical management, parent perspective

## Abstract

**Background:**

Parents are intricately involved in managing their child’s lower respiratory tract infection (LRTI). While medical and physiotherapy management of childhood respiratory infections has been studied, little is known about parents’ perspectives on the condition and its care.

**Objectives:**

The aim of this study was to explore the experiences and challenges of parents of children with LRTI concerning medical and physiotherapy management.

**Method:**

Parents of children under 3 years with LRTIs were invited to participate in a qualitative exploratory study. Semi-structured interviews were conducted. Each interview was coded, and deductive thematic analysis was used to identify themes.

**Results:**

Data saturation was reached after 10 interviews and confirmed after 12 interviews. The mean ages of participants and their children were 34.3 (standard deviation [s.d.] ± 4.9) years and 18.8 (s.d. ± 6.5) months, respectively. The codes that emerged formed sub-themes within a main theme, which was labelled ‘It is tough taking care of a sick child’. The sub-themes identified were ‘personal challenges’, ‘treatment options’ and ‘parent understanding’.

**Conclusion:**

Parents still felt ineffective and stressed about taking care of their child’s LRTI, despite the integral role that they play in the management of their child’s respiratory condition. Timely access to a multidisciplinary team of paediatric healthcare professionals who practice family-centred care positively influences the experience of caring for a sick child.

**Clinical implications:**

Understanding parent perspectives will help healthcare professionals enhance a family-centred care approach, leading to better health outcomes.

## Introduction

Lower Respiratory Tract Infections (LRTIs) are a substantial cause of hospitalisation among infants and children in South Africa and globally (Kyu et al. 2016; Zar et al. 2020). A study by Mirkarimi et al. (2020) found that half of the children who required hospital admission for LRTIs were below 1 year of age. Despite improvements in management, LRTIs remain a significant concern in South African children under 5 years (Corten & Morrow 2020; Zar et al. 2020).

Lower Respiratory Tract Infection is an overarching term for infections, which are usually viral or bacterial, which involve the respiratory tract below the larynx. This includes bronchopneumonia, pneumonia, bronchiolitis and bronchitis (Boloursaz et al. 2013). The most common cause of LRTIs, particularly bronchiolitis, is the respiratory syncytial virus (RSV) (Nair et al. 2010). Children hospitalised with RSV-associated pneumonia frequently have co-occurring pneumococcal infections (White et al. 2016). Most children develop antibodies against RSV by 3 years of age, but this does not imply life-long immunity (Kutsaya et al. 2016).

Respiratory viruses cause harm to the epithelium of conducting airways and distal alveoli, where gas exchange occurs, resulting in excess mucus production because of inflammation and necrosis (Pham et al. 2020). Coughing, wheezing, increased work of breathing, nasal congestion, rhinorrhoea and fever are common features of LRTIs (Pham et al. 2020). Infants who still rely on nose-breathing may have difficulty feeding.

The diagnosis of LRTIs is usually based on clinical presentation (Mirkarimi et al. 2020). Rapid antigen and the multiplex polymerase chain reaction (PCR) tests are used to detect RSV (Newman et al. 2020). Medical management of LRTIs is determined by the severity of the patient’s symptoms. Oxygenation and hydration status are important factors to consider (Mallory et al. 2003). Nebulising with 3% saline is effective and is common practice (Hsieh et al. 2020; Ralston et al. 2014).

Infants with LRTIs frequently require chest physiotherapy (Morrow 2019) to facilitate the removal of excessive secretions (Volsko 2013) and improve breathing exertion and gaseous diffusion (De Boeck et al. 2008). Chest physiotherapy comprises a combination of nebulisation, postural drainage, percussions (De Boeck et al. 2008) and nasopharyngeal suctioning (Argent & Morrow 2004) to remove loosened secretions (Morrow & Argent 2008). Younger children who are unable to blow their nose and have a weak cough may benefit from suctioning.

Mobilisation in the form of play (Morrow 2019) and the active cycle of breathing technique (Fink 2007) can be used in combination with manual airway clearance treatment. Treatment plans should be individually determined depending on each child’s needs. Routine chest physiotherapy is unnecessary and has the potential to be costly and distressing for the child and caregiver (De Boeck et al. 2008; Krause & Hoehn 2000). Parents of children with cystic fibrosis are integrally involved in the continuation of chest physiotherapy treatment. This therapy involvement creates feelings of pressure and doubt, but also gives the parents the opportunity to facilitate a positive impact in their child’s life (Andrews, Smith & Cox 2020). The literature on the psychological effect of chest physiotherapy on both the child with an LRTI and their parents’ perspective towards it is limited. Educating parents on the various aspects of an LRTI is an effective method for preventing further infections and managing their expectations regarding respiratory management (Taylor, Kwan-Gett & McMahon 2003).

The stress of caring for a sick child can be mitigated by adopting the family-centred care approach. Family-centred care is a theoretical framework including parents in the planning of their child’s treatment regime (Shields et al. 2007). Family-centred care principles have shown that when parents are involved, there is a higher level of engagement from the child in treatment activities (Shields et al. 2007). Additionally, the parents’ anxiety levels are directly linked to those of the child (Cameron, Bond & Pointer 1996; Sweeney & Wilson 2023). Effective communication can decrease stress levels, improve participation and autonomy in daily activities. The healthcare team’s attitudes and behaviours can affect the parents’ concerns and level of engagement, influencing them in both positive and negative ways (Power & Franck 2008). Each family unit should be treated individually by a cohesive healthcare team. In children under the age of 5 years, parents are integrally involved in all aspects of care and management.

This study aimed to explore the experiences and challenges faced by parents of children aged under 3 years old, with LRTIs, regarding medical and physiotherapy management. The findings might assist the healthcare team to advocate for a more tailored, family-centred care approach to the management of children with LRTIs.

## Research methods and design

This research study utilised a qualitative, exploratory descriptive approach. Individual, semi-structured interviews were conducted until data saturation was reached, defined as the point where no new themes or insights emerged from subsequent interviews. Participants were recruited from two different facilities – an inpatient paediatric unit at a private hospital, and an outpatient private practice setting. Both sites treat children between birth and 12 years of age and are in Gauteng (Foot 2023).

Participants were parents of children under 3 years of age who had a confirmed diagnosis of LRTI. Purposive sampling was used, and parents (mothers and fathers) of children referred for chest physiotherapy who met the specified inclusion criteria were invited to take part in the study. Children who had undergone at least one chest physiotherapy session, which was observed by the parent being interviewed, were included (Foot 2023). Parents of Neonatal Intensive Care Unit (NICU) and/or Paediatric Intensive Care Unit (PICU) patients were excluded, as their experiences differ significantly because of the higher emotional burden associated with intubation and critical care. The study focused specifically on non-intubated patients. Patients were not included if the researcher was the sole treating physiotherapist to avoid bias and coercion.

Ethical clearance and permission from the clinical sites were obtained. Potential participants were identified by the researcher at both research sites, in hospital and private practice settings, and were invited to participate in the study. Potential participants were provided with information regarding the study procedure, an explanation of the roles and responsibilities of both the parent participant and the researcher, who was the principal investigator and conducted all the interviews. A statement informing parents that they could withdraw from the study at any time without facing any consequences was included. This information was offered in the participant’s preferred language. A minimum of 24 h was given for parents to decide whether they wished to participate. If they agreed to participate, signed consent was obtained for both participation in the study and for the audio-recording of the interview (Foot 2023).

Semi-structured interviews were conducted at a mutually convenient time, either in the child’s own hospital room or a private room within the outpatient department. The participant completed a demographic questionnaire, which included questions on their background and the birth history of the child. A translator was available to translate from English to Sotho, Zulu, Tswana and Afrikaans, if required. All patients were comfortable to conduct the interview in English; thus, no translator was needed.

The semi-structured interviews were conducted by using an interview guide (Online Appendix 1) by the principal researcher (Danielle Foot) and were recorded on iPhone Audio-Note application. Two pilot interviews were conducted to ensure the recording was clear and to confirm that the questions were understood by participants and elicited the desired information. The pilot interviews were included for analysis after the principal researcher (Danielle Foot) and supervisor (Joanne L. Potterton) reviewed them, and no changes were deemed necessary (Foot 2023).

Participants could choose in which language they wanted to be interviewed. The in-person interviews lasted approximately 30 min. During the interview, short notes were made documenting reactions or relevant context. To minimise the paper trail, these notes were recorded on the back of the completed demographic questionnaire. Data saturation was achieved after 10 interviews when no new information emerged during the interviews (Foot 2023). This saturation was confirmed by adding another two interviews to ensure no new information emerged.

Once their interview was completed, participants were asked if they had any additional concerns or questions. Refreshments and various appropriate games and crafts were available to keep the children busy if they were present. All coronavirus disease 2019 (COVID-19) precautions and safety measures were followed. The principal researcher (Danielle Foot) anonymised and transcribed the interview verbatim. The audio recordings were deleted from the iPhone Audio-Note application, and the transcriptions, along with the demographic data and interview notes, were stored on a password-protected computer. Data will be stored and then deleted 2 years after publication (Foot 2023).

Demographic data were summarised and analysed using Excel. To analyse the interviews, a manual coding system was created. Primary codes were assigned using a deductive approach. A second-order category was developed from these codes (Punch 2014). Thematic analysis enabled themes to be developed from the categories. Relevant quotes were obtained from interviews to illustrate themes identified. Coding was carried out separately by the principal researcher (Danielle Foot) and supervisor (Joanne L. Potterton) and compared; no discrepancies were found (Foot 2023). This ensured intercoder reliability, adding to the overall consistency of the study.

To ensure trustworthiness, the principles outlined by Lincoln and Guba were applied (Lincoln & Guba 1985). The short notes made during the interviews contributed to the credibility of the findings. Thick descriptions of the two settings and characteristics of participants contributed towards ensuring transferability. Dependability was achieved by using a consistent interview guide (Online Appendix 1) across interviews. The use of raw quotes extracted from the interviews contributed to the authenticity of the themes (Colorafi & Evans 2016; Lincoln & Guba 1985). Ontological assumptions are identified and acknowledged. The principal researcher is a young physiotherapist without children of her own at the time of conducting the interviews. However, she treats multiple paediatric patients and interacts with these parents daily (Foot 2023).

### Ethical considerations

The Human Research Ethics Committee of the University of the Witwatersrand granted ethical clearance (reference number: M210722). The study obeyed the ethical principles of justice, beneficence, autonomy and non-maleficence in accordance with the Declaration of Helsinki (World Medical Association 2013). A distress protocol was available if needed. If participants became distressed, they would be referred to a counselling psychologist who agreed to be available if required. However, this was not needed. Approval was obtained from the sites where the research was conducted. Written consent was secured for both participation in the study and for the audio recording of interviews (Foot 2023). Anonymity was achieved by assigning codes to each participant; no identifying information was used or recorded. Participants were assured that they may withdraw or choose not to answer a question without a formal reason and with no repercussions for the participant or patient.

## Results

Data saturation was achieved after 12 interviews. Table 1 summarises the demographic information of the participants. All participants were the mothers of the children with a respiratory condition and were married. The mean age was 34.3 ± 4.9 years, and there was an equal number of participants from the inpatient and outpatient settings.

**TABLE 1:** Demographic information of participants (*N* = 12).

Demographic data	*n*	Mean ± s.d.	Median	IQR
Age (years)	-	34.2 ± 4.9	33.5	30.5–39.0
Mother	12	-	-	-
Tertiary Education	11	-	-	-
Attended outpatient setting	6	-	-	-
Attended in-hospital setting	6	-	-	-

s.d., standard deviation; IQR, interquartile range.

The diagnosis of each child is documented in Table 2. The majority of the children were diagnosed with bronchiolitis (*n* = 8). Seven of the patients were diagnosed with RSV, and only one child had influenza A. In four cases, no specific pathogen was specified.

**TABLE 2:** The diagnosis of each child is documented as reported by the mother (*N* = 12).

Diagnosis	*n*
Bronchiolitis	8
Pneumonia	2
Bronchopneumonia	2
RSV detected	7

RSV, respiratory syncytial virus.

Table 3 summarises the background information about the child and hospital admissions. The mean age of the children was 18.8 ± 6.5 months, six children had siblings, and nine attended crèche. Eight children had previous hospital admissions for a variety of illnesses. Six children had received chest physiotherapy prior to this illness. One child was born prematurely but was not admitted to the NICU and/or PICU.

**TABLE 3:** Background information pertaining to the child and hospital admissions (*N* = 12).

Additional child information	*n*	Mean ± s.d.	Median	IQR
Age (months)	-	18.8 ± 6.5	16.5	8.5–22.5
Has siblings	6	-	-	-
Attends crèche	9	-	-	-
Had a previous hospital admission	8	-	-	-
Previous chest physiotherapy	6	-	-	-
Premature birth (< 37 weeks)	1	-	-	-

s.d., standard deviation; IQR, interquartile range.

### Thematic analysis

**FIGURE 1:**
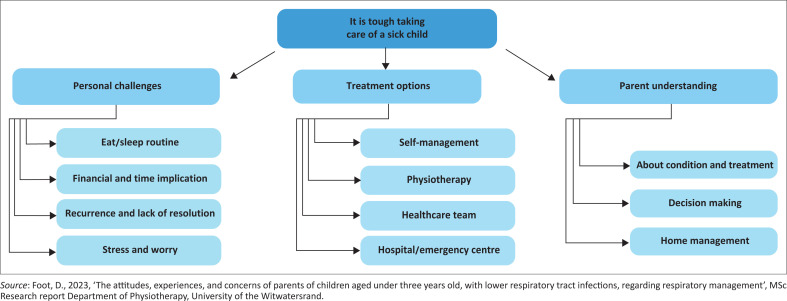
Thematic analysis.

The codes that emerged formed sub-themes and one overarching theme. The main theme was ‘it is tough taking care of a sick child’. Figure 1 illustrates the development of the theme and sub-themes from the codes. Three sub-themes were identified, namely, personal challenges, treatment options and parent understanding.

#### Personal challenges

**Eat and sleep routine:** The difficulty of ensuring that a sick child eats adequately and maintains his sleeping patterns emerged in 11 of the interviews:

‘[*I*]t’s been a bit of a challenge with regards to his sleeping, his eating, and drinking. With all the slime and the breathing problems, he has not really wanted to eat or drink …’ (Participant 2, mother, 31 years)

Financial and time implications: Over half of the participants (*n* = 7) highlighted the financial and time-related challenges associated with caring for a child with an LRTI. Mothers expressed how trying to balance caring for a sick child and working was a challenge, potentially impacting their ability to fulfil both roles:

‘[*W*]orking and looking after him is, is a 24 -7 thing, um, literally not leaving them alone.’ (Participant 5, mother, 39 years)

‘It’s a lot of work to do because you have to nebulise, you have to give them antibiotics, then you have to do the physio [*physiotherapy*]. So, it’s quite a lot.’ (Participant 7, mother, 29 years)

Participants spoke about financial strain caused by their child’s illness. Out-of-hospital visits and medication costs depleted their medical aid savings early in the year:

‘Very expensive. Our medical aid was depleted in June or July. So since then, we have to pay everything cash when we go to the doctor or yeah, and medicine and then physio [*physiotherapy*] as well, so it’s quite rough.’ (Participant 4, mother, 32 years)

**Stress and worry:** Participants experienced stressful emotions while taking care of their child with an LRTI. The things that worried mothers most were when their child was breathless and the fact that their children were still so young and vulnerable:

‘[*I*]t’s been fairly worrying given that she is so small and was born prematurely … so it is a little bit of a worry, especially because it’s now the start of RSV [*respiratory syncytial virus*] season …’ (Participant 9, mother, 37 years)

‘I was panicking.’ (Participant 8, mother, 31 years)

#### Treatment options

**Self-management:** The majority of participants had a range of home treatments they utilised before seeking professional assistance. Those who had previously experienced a ‘sick child’ episode noted that this prior experience helped them better manage the current situation:

‘Given that I’m a third-time mother and I’ve had a baby with RSV [*respiratory syncytial virus*]. I think I’ve handled it quite well … I was a little bit more worried this time, given that she is so small.’ (Participant 9, mother, 37 years)

‘She is the second born, um, I’ve known prior to now how to handle the situation. When she coughs, I always try to listen if it’s a wet or a dry cough … having a fever with it … What colour is the mucus … I’ll know whether to take her to the GP [*general practitioner*] or whether to take her to the paediatrician … we can’t treat her as effectively at home … I know what to expect. I know what is next … nothing shocks me anymore … I have seen it all …’ (Participant 12, mother, 30 years)

Parents experiencing a child with an LRTI for the first time did not have the same confidence:

‘So, because I was panicking, I ended up just touching and going, trying this spray, trying this medication.’ (Participant 8, mother, 31 years)

**Physiotherapy:** Participants had differing opinions about whether they preferred to be present during their child’s physiotherapy session or not. Seven participants reported that the child appeared significantly calmer when they were present during the physiotherapy session. One reported that she could not conclude whether it was better to be present during the treatment or not, and four stated that they chose not to be present as it upsets both the mother and their child:

‘[*H*]e realised that she’s not there to hurt him and I’m not going anywhere. So, I think he felt more at ease that I was there and he wasn’t being hurt at all.’ (Participant 1, mother, 27 years)

‘It’s calming for me to be with him …’ (Participant 5, mother, 39 years)

When asked about their experience with physiotherapy, the majority of parents (*n* = 11) reported that the experience was positive and beneficial for the child. Most mothers were initially concerned, then reassured once they had seen what treatment entailed:

‘General experience very, very happy … I think the physio [*physiotherapy*] made the biggest part of the difference for the hospital stay … it didn’t bother me that I was not present … Positive and it’s helped him to be able to breathe [*referring to physiotherapy*].’ (Participant 2, mother, 31 years)

‘Of course, the process of the physio [*physiotherapy*] is very, it’s very painful to the mom, but it has to be done. So, it’s not something I can say I’m concerned about. It’s just as a mom, I don’t want to see it … I just liked the results, but the process is it’s a bit painful to witness …’ (Participant 8, mother, 31 years)

Only one mother reported that it was traumatic, and she never wanted to go through the experience again:

‘So, I think we are both equally traumatised … she was screaming the whole time … I can’t go through it again. It’s just, it’s terrible seeing my child like that … So, whilst I understand she needs to have the physio [*physiotherapy*], it helps her. I mean, I, I can’t think that a child screaming like that, that it ultimately helps her.’ (Participant 6, mother, 42 years)

Mothers agreed that suctioning was the worst aspect of treatment but appreciated its value:

‘I think that is the most important [*referring to the suctioning*]. So, I think the suctioning is quite a traumatic experience. I think even for me, I was a bit traumatised. But it’s quick. And I think she realises afterwards, she feels better.’ (Participant 11, mother, 39 years)

Mothers expressed the need for healthcare professionals, including physiotherapists, to be trained in paediatrics. Overall, they were satisfied with the feedback received after treatment:

‘My experience up to now was that there was only one specific person who actually knew what she was doing. You are either meant to work with children or you are not. So, if you don’t know how to work with babies, you are at the wrong place.’ (Participant 5, mother, 39 years)

**Healthcare team:** The discussion about practitioner visits focused on the specialisation of care. The majority of mothers would rather take their child to a paediatrician rather than a GP (general practitioner) as they perceive the paediatrician as a clinical expert:

‘GPs don’t specialise in kids that much … they don’t know how to treat it … So that’s a very big problem … try to get to the paediatrician as soon as possible.’ (Participant 12, mother, 30 years)

Participants emphasised the need for improved communication regarding treatment plans and investigation results from the healthcare professionals. They also expressed a desire for medical practitioners to allocate more time for interactions and to listen more attentively to their concerns:

‘So, I do think if they actually just spend two minutes, just stop, just explain what’s happening.’ (Participant 6, mother, 42 years)

**Hospital or emergency centre:** The majority of mothers felt at peace knowing that their child was receiving professional treatment in the hospital:

‘Yes, definitely gave you some peace of mind [*being admitted to hospital*] … just to know that she is in good hands, and they are looking after her, so definitely peace of mind compared to being at home …’ (Participant 9, mother, 37 years)

#### Parent understanding

**About the condition and treatment:** Participants were able to express their understanding of what was wrong with their child:

‘Well, right now we do know that he is sick with a virus that affects his lungs. Um, well, he’s got a few viruses that it is affecting his lungs, his ears, and so forth. So, with the phlegm that is also building up, it makes it difficult … for him to breathe. So, the nebulising, the physio [*physiotherapy*] obviously helps to loosen the phlegm and for him to get it out, um, so that he can breathe … easier.’ (Participant 5, mother, 39 years)

Most mothers (*n* = 7) still wanted more information about the disease progression, the indications for treatment, and what outcomes could be expected:

‘I want to know, I want to understand what’s wrong, what we are, what we are trying to fix and how the treatment that they are prescribing is fitting into, getting her, getting her healthy.’ (Participant 6, mother, 42 years)

**Decision-making:** When asked about their involvement in the decision-making process regarding their child’s treatment, responses were mixed. Two out of the 12 mothers stated that they felt they knew their child best and valued being involved in the decision-making process:

‘Um, but everything explained and, you know, also I was involved with even nebulising her and … everything. So, I felt very involved.’ (Participant 9, mother, 37 years)

‘They ask us are you comfortable … we feel involved either me or the father. We were consulted.’ (Participant 12, mother, 30 years)

In contrast, some caregivers were happy for the healthcare team to take the lead in the decision-making:

‘They just say this is what’s going to happen. Um, and to be honest, I’m okay with that because I know that is what needs to happen for him to get better.’ (Participant 1, mother, 27 years)

**Home management:** The majority of participants raised concerns about home management after discharge:

‘[*I*]s there anything like at home that we could do that could maybe help that we’re not doing? Cause at the moment we just doing like medicine-based things we don’t really do physio [*physiotherapy*], like, you know, like how you would tap his back or something like that?’ (Participant 7, mother, 29 years)

## Discussion

This study focused on exploring the experiences and concerns of parents of children under 3 years of age with LRTIs regarding the respiratory management of their child. The participants of this study were the mothers of the children (Foot 2023). Typically, women serve as the primary caregivers for their ill children while also managing their usual daily tasks and work obligations. This dual responsibility can contribute to maternal burnout, which may be alleviated by involving fathers and other family members in the caregiving process (Rafii, Oskouie & Shoghi 2014; Vieira & Cunha 2020), especially when children are ill with common childhood illnesses such as RSV.

Bronchiolitis caused by RSV was diagnosed in more than half of the children (*n* = 7). Respiratory Syncytial Virus is most prevalent between February and March in Gauteng, South Africa (White et al. 2016). In this study, children tested positive for RSV as early as December 2021 (Foot 2023). This early spike can be attributed in part to the COVID-19 pandemic and subsequent lockdowns. This led to decreased exposure to RSV, which in turn led to decreased RSV population immunity; this concept has been termed ‘RSV immunity debt’ (Billard & Bont 2022; Cohen et al. 2021). After the easing of lockdown regulations, there was a significant increase in exposure, leading to surges occurring out of season, particularly in young children (Foot 2023).

The mean age of patients in this study was 18.8 months. In a study by Mirkarimi et al. (2020), half of the children who required hospital admission were below 12 months of age (Mirkarimi et al. 2020). This is similar to our study, where 50% of the children who were admitted to the hospital were below 1 year of age. Children of this age require constant care; this can be challenging for the caregivers when the children are ill and demands increase.

### Personal challenges

Most participants commented on how the disrupted sleeping habits of the sick child impacted negatively on the entire family. An LRTI impairs a child’s ability to breathe and cough effectively, which in turn negatively affects their sleep and eating habits (Pham et al. 2020).

Over half of the participants emphasised the time demands and financial strain of caring for a child with an LRTI (Foot 2023). A recent study found that in low-income households, the financial burden of an RSV illness episode amounted to 32% of the total monthly household income (Baral et al. 2020). This financial statement validates caregivers’ concerns about the expenses involved in caring for an ill child and would be of even greater concern to parents from low socio-economic backgrounds who were not represented in this study.

Although this study did not record whether the participant was employed or not, most mothers reported that looking after a sick child and working was challenging. There is a paucity of research on working mothers and the challenges they experience. The difficulty of dual responsibility is supported by a study by Vo et al. (2014), where 88.04% of caregivers reported how difficult it was to navigate daily tasks and responsibilities when caring for their sick or hospitalised children.

Consistent with previous research (Halls et al. 2017), parents of younger children tend to be more concerned about their child’s condition because of their limited ability to communicate and the potential for rapid deterioration. Participants mentioned that their treatment strategies during this stressful event were often ineffective and an emotional response to fear and anxiety, rather than a well-tailored management plan (Foot 2023). Parents are more cautious when their children are younger and are more likely to consult a healthcare provider (Halls et al. 2017).

### Treatment options

Parents of children with cystic fibrosis are involved with the continuation of chest physiotherapy at home themselves, nebulising and percussions specifically (Andrews et al. 2020). Although there are currently no guidelines for managing LRTIs at home, most participants first attempted a combination of nebulising, medication and at-home suctioning devices before consulting a healthcare professional. However, they still lacked confidence and felt ineffective in their own approach and skills. Nebulisation at home with 3% saline (Hsieh et al. 2020) and the use of superficial nasal suctioning at home (Joshi, Parihar & Singh 2019) have both been described as effective home management strategies used by caregivers of children with LRTIs.

Mothers who had previously experienced a similar sickness episode felt more confident and prepared to manage the current situation, drawing on their past experiences (Foot 2023). Halls et al. (2017) state that a previous sickness experience was a guiding key factor enabling parents to judge the severity of their child’s illness.

When mothers did consult the healthcare team, they reported that they had conflicting feelings about being present during physiotherapy sessions. The majority of mothers said they would prefer to be present as it calms the child (Foot 2023). No literature was found on parental experiences of physiotherapy or witnessing the suctioning process; thus, the personal preference of the parent should prevail. Most participants appreciated the value of chest physiotherapy despite initial reticence. In our study, participants commented that watching their child being suctioned was traumatic, but that this is the technique that was most effective in improving the child’s clinical symptoms. This is the first study to identify the experience of parents witnessing the suctioning process; the findings can be used by physiotherapists to prepare parents for what they can expect during their child’s chest physiotherapy treatment and contribute to the current gap in the literature.

A related study by Andrews et al. (2020) investigated parents’ perspectives regarding their involvement in the respiratory care of their children with cystic fibrosis and found that parents can positively impact their child’s treatment experience. Providing parents with comprehensive education about chest physiotherapy treatment and its significance was beneficial for them (Andrews et al. 2020). This supports the findings of our study, where caregivers felt equipped after clarification or demonstration of the treatment technique (Foot 2023).

In this study, all mothers chose to consult a healthcare professional despite attempting their own treatment regimens (Foot 2023). This finding is not supported by the study from Hay et al. (2019), where only 8% of participants preferred to consult a healthcare professional for a paediatric respiratory infection; however, the recruitment strategy for the current study only included parents whose children were already receiving physiotherapy, which would bias this sample. Mothers commented that they would prefer to consult a paediatric expert, given that their child was still so young. Participants expressed their preference for a physiotherapist with experience and skill in paediatric treatment techniques rather than a generalist in the profession. Mothers stated that they prefer management and treatment tailored for their needs (Boelsma et al. 2021).

Mothers were not satisfied with the limited time spent by practitioners to actively listen to them and make an individualised clinical diagnosis and plan treatment based on their needs. This includes feedback on results and the severity of the condition. Rushed consultations and insufficient feedback are also mentioned by Hay et al. (2019) in their study performed in Bristol, United Kingdom, which investigated the proportion of parents seeking primary care help when their child develops a respiratory tract infection. A sick child admitted to the hospital is a stressful experience for any caregiver (Loyland et al. 2019); however, this study indicates that some caregivers felt reassured when their child was admitted to the hospital and receiving the care they needed.

### Parent understanding

Mothers were able to identify their child’s symptoms, but they were not able to explain the underlying pathology. Halls et al. (2017) identified a similar gap in knowledge among parents regarding their child’s respiratory diagnosis. Participants emphasised the need for healthcare professionals to provide information regarding the rationale behind performing certain tests or treatment regimes, the indication for hospital admission, potential medication side-effects, and an explanation for deteriorating symptoms (Foot 2023). These issues should serve as an educational guide for healthcare professionals wishing to follow a family-centred care approach.

Parents play an integral role in managing their child’s respiratory illness. Participants emphasised that they understood their child the best and stressed that the healthcare professionals respected their opinions and insights (Foot 2023). This phenomenon of a ‘mother knows best’ correlates with a similar study (Halls et al. 2017). Halls et al. (2017) investigated the parent perspective regarding the management of their child with an LRTI, including the use of antibiotics. In line with family-centred care principles, primary caregivers are key participants in their child’s treatment process (Shields et al. 2007). Health education can equip parents to make informed decisions and feel empowered in the care of their child (Lopes & Dixe 2012). This education should be reinforced, and detailed individual discharge plans should be developed in consultation with the parents.

### Strengths and limitations

To our knowledge, this is the first study to explore the perspective of parents regarding the medical and physiotherapy management of their child with an LRTI conducted in South Africa. It is also the first study to include parents’ perspectives about being present during physiotherapy treatment, specifically when their child was being suctioned. The research was carried out in Gauteng, South Africa, within private in-hospital and outpatient settings, allowing the results to be generalised to both populations (Foot 2023).

One of the limitations is that since participants were only recruited from private settings, the findings only reflect the perspectives of parents who access private healthcare. This excludes the majority of South Africans who rely on the public healthcare system, where resources, care pathways and family experiences may differ substantially. Findings cannot therefore be generalised to the public setting.

All children were under the age of 3 years and received nasopharyngeal suctioning as part of chest physiotherapy treatment. Therefore, parents of older children might express different attitudes and/or experiences and/or concerns. All participants were the mothers of the child; therefore, these findings may not be generalisable to other caregivers such as fathers, grandparents or other primary caregivers.

### Implications for clinical practice

The aim of this study was to explore the attitudes, experiences and concerns of parents of children regarding the medical and physiotherapy management. These parent perspectives should serve as a foundation for guiding treatment goals. Parents must be allowed to decide whether or not they wish to be present during physiotherapy treatment sessions. Parents prefer healthcare professionals who are experts in paediatrics and who communicate openly and acknowledge that ‘the mother knows best’ (Foot 2023). Healthcare professionals should therefore explain techniques beforehand and offer reassurance, especially when using procedures that may appear distressing to parents, such as suctioning.

Healthcare professionals should be cognisant of the financial and time constraints faced by parents when planning a treatment programme. Supplementary educational material in the form of an advice pamphlet or educational video on respiratory treatment should be considered when advising the caregivers on the various treatment options and home management strategies available (Foot 2023).

## Conclusion

This qualitative research study explored the experiences and challenges of parents regarding the management of their child with an LRTI. Mothers are the primary caregivers when their children are sick and try various home treatments before seeking medical attention for their child. Most mothers reported feeling ineffective in their management abilities and chose to seek professional advice. However, those who had experienced a previous sickness episode were more self-assured (Foot 2023).

The majority of mothers expressed satisfaction with the physiotherapy treatment and the feedback provided afterwards and preferred to be present during the sessions. They expressed the need for a good team of paediatric specialists and stressed the importance of good communication and education. Most mothers felt reassured when their child was admitted to a hospital, even though it disrupted the child’s routine.

As the patient population of this study only included the private sector, future research could be conducted to include the public sector as well. The involvement of the father in the management of a child with an LRTI could also be investigated.

The overarching theme of this qualitative study was ‘It is tough taking care of a sick child’. Healthcare professionals should consider this perspective when developing a personalised respiratory management plan for each child in collaboration with their families (Foot 2023). The findings of this study highlight the need for a holistic family-centred care approach that incorporates a parental perspective into paediatric respiratory care in managing children with LRTIs.
